# Supplementary material for: Probing the symmetry breaking of a light–matter system by an ancillary qubit
Source: Nat Commun. 2023 Jul 20;14:4397. doi: 10.1038/s41467-023-40097-0 (PMC10359332; doi:10.1038/s41467-023-40097-0)
Supplement: Supplementary file 1 — Supplementary Information [file 41467_2023_40097_MOESM1_ESM.pdf]

Supplementary Information for "Probing the symmetry breaking of a light–matter system by an ancillary qubit"

Shuai-Peng Wang,[1,2] Alessandro Ridolfo,[3] Tiefu Li,[4,1,5,*] Salvatore Savasta,[6,†] Franco Nori,[7,8] Y. Nakamura,[9,10] and J. Q. You[2,1,‡]

[1] *Quantum Physics and Quantum Information Division, Beijing Computational Science Research Center, Beijing 100193, China*
[2] *Interdisciplinary Center of Quantum Information, State Key Laboratory of Modern Optical Instrumentation, and Zhejiang Province Key Laboratory of Quantum Technology and Device, School of Physics, Zhejiang University, Hangzhou 310027, China*
[3] *Dipartimento di Fisica e Astronomia, Università di Catania, 95123 Catania, Italy*
[4] *School of Integrated Circuits, and Frontier Science Center for Quantum Information, Tsinghua University, Beijing 100084, China*
[5] *Beijing Academy of Quantum Information Sciences, Beijing 100193, China*
[6] *Dipartimento di Scienze Matematiche e Informatiche, Scienze Fisiche e Scienze della Terra, Università di Messina, I-98166 Messina, Italy*
[7] *Theoretical Quantum Physics Laboratory, RIKEN Cluster for Pioneering Research, Wako-shi, Saitama 351-0198, Japan*
[8] *Physics Department, The University of Michigan, Ann Arbor, Michigan 48109-1040, USA*
[9] *RIKEN Center for Quantum Computing (RQC), Wako, Saitama 351-0198, Japan.*
[10] *Department of Applied Physics, Graduate School of Engineering, The University of Tokyo, Bunkyo-ku, Tokyo 113-8656, Japan*

ADDITIONAL EXPERIMENTAL DETAILS

The dependency of the resonance frequency of the lumped-element resonator ω_r on the external flux bias $\delta\Phi_{\rm ext}$ around the optimal point can be approximately described as

$$\omega_r(\delta\Phi_{\rm ext}) = \frac{\omega_r(0)}{\sqrt{1 + D\cos\theta\,|\delta\Phi_{\rm ext}|}}\,, \qquad (1)$$

where the constant $D = -0.18$ and is determined by tuning the fitting curve of ω_{01} in Fig. 2c of the main text when $\omega_r(0)$ is fixed.

Supplementary Fig. 1 shows the the experimental setup.

Supplementary Fig. 2 displays the bare and normalized reflection spectra without the fitting curves of Figs. 2a and 2b in the main text. The abrupt changes in the transmission background around 5.6 GHz and 11.9 GHz can be seen in the bare spectra in Supplementary Figs. 2a and 2b.

Supplementary Fig. 3 displays the calculated excitation spectra ($\omega_{0,n}$) of the total system (the deep-strongly coupled qubit–resonator system plus the Xmon). The observed transitions (ω_{01}, ω_{02} and ω_{03}) in Fig. 2 of the main text correspond to $\omega_{0,1}$, $\omega_{0,3}$ and $\omega_{0,6}$. The sideband transitions in Fig. 2a of the main text correspond to (from inside to outside) $\omega_{4,18} \approx \omega_{04,X} - (\omega_{01} + \omega_{01,X})$, $\omega_{5,20} \approx (\omega_{02} + \omega_{03,X}) - \omega_{02,X}$, $\omega_{5,16} \approx (\omega_{04} + \omega_{01,X}) - \omega_{02,X}$ and $\omega_{5,15} \approx \omega_{05} - \omega_{02,X}$, where $\omega_{n,m} \equiv \omega_{0,m} - \omega_{0,n}$ and $\omega_{0n,X}$ denotes the dressed excitation energy levels of the Xmon. The observation of these high-order sideband transitions near the band edge is a surprise, which may demand a further theoretical study.

THEORY

Theoretical description

We consider a deep-strongly coupled (DSC) system constituted by a flux qubit coupled inductively to a lumped-element resonator through a shared Josephson junction. This system can display a *photonic vacuum symmetry breaking* [1]. Here we demonstrate that the presence of such symmetry breaking can induce the breaking of parity selection rules of an Xmon artificial atom interacting dispersively with the ultrastrongly coupled system via the lumped-element resonator. This selection-rule breaking can then be probed by applying a driving field on the coupled Xmon.

The total Hamiltonian for our three-component system can be written as

$$H_{\rm tot} = H_s + H_X^{(4)} - g'(a - a^\dagger)(b - b^\dagger)\,, \qquad (2)$$

where H_s is the Hamiltonian of the DSC qubit–resonator system in the main text, and

$$b = \sum_{n=0}^{3} \sqrt{n+1}\,|n\rangle\langle n+1| \qquad (3)$$

is the annihilation operator for the Xmon modeled here as a four-level artificial atom (qudit). The bare Xmon as a qudit is

$$H_{\rm X}^{(4)} = \sum_{n=0}^{3} \varepsilon_n |n\rangle\langle n|\,, \qquad (4)$$

where we choose $\varepsilon_0 = 0$ for convenience.

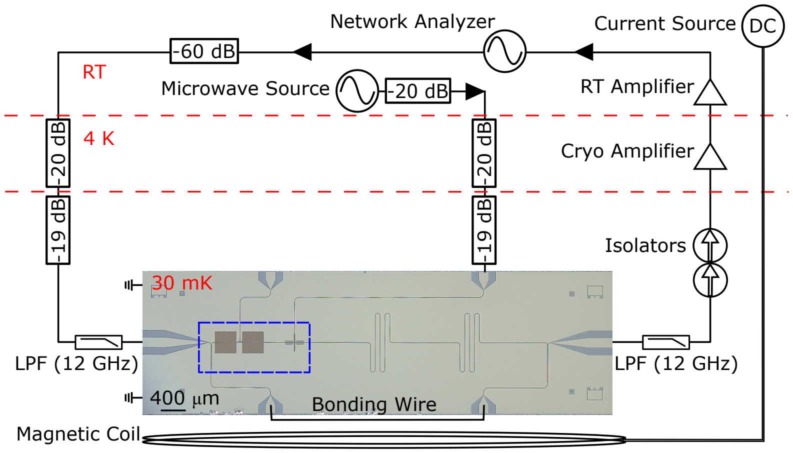

Supplementary Fig. 1. Schematic of the experimental setup. Figure 1a in the main text is the area denoted by the blue rectangular box. RT: room temperature. Cryo: cryogenic. LPF: low pass filter.

Owing to the parity symmetry, two-photon transitions in the bare Xmon artificial atom are forbidden. Thus, an observation of the two-photon transition represents a signature of induced breaking of the selection rules in the Xmon coupled to the qubit–resonator system. Below we show that the two-photon transition rate can be directly calculated using second-order perturbation theory.

Let us consider a single-tone external drive exciting the Xmon,

$$H_1(t) = (\Omega/2)(Y^- e^{-i\omega_d t} + Y^+ e^{i\omega_d t}) , \tag{5}$$

where

$$Y^+ = -i \sum_{j<k} \langle E_j|(b - b^\dagger)|E_k\rangle \, |E_j\rangle\langle E_k| , \tag{6}$$

with $|E_j\rangle$ being the eigenstates of the Hamiltonian (2), which are ordered so that $j < k$ if $\omega_j < \omega_k$.

We choose system's ground state $|E_0\rangle$ as the initial state. The first-excited state of the system corresponds to the dressed first-excited state of the DSC qubit–resonator system. The second-excited state of the system corresponds to the dressed first-excited state of the Xmon. The transition rate from $|E_0\rangle$ to $|E_2\rangle$ can be written as

$$W_2(\omega_d) = 2\pi \left|\mathcal{T}_{0,2}\right|^2 \delta(\omega_{0,2} - 2\omega_d) , \tag{7}$$

where $\omega_{j,k} = \omega_k - \omega_j$, and

$$\mathcal{T}_{0,2} = \Sigma_k \frac{V_{0,k} V_{k,2}}{\omega_{0,k} - \omega_d} . \tag{8}$$

Here, the matrix elements of the perturbation potential are

$$V_{j,k} = \frac{\Omega}{2} \langle E_j|Y^+|E_k\rangle . \tag{9}$$

The losses of the system components can be included by converting the Dirac delta function in Supplementary Eq. (7) into a Lorentzian:

$$\delta(\omega_{0,2} - 2\omega_d) \rightarrow \frac{1}{2\pi} \frac{\Gamma_{0,2}}{(\omega_{0,2} - 2\omega_d)^2 + \Gamma_{0,2}^2/4} . \tag{10}$$

The definition of the loss rates as $\Gamma_{2,0}$ can be found in the next section.

The two-photon Xmon power spectrum can be obtained as

$$\langle Y^- Y^+\rangle = \mathrm{Tr}[\rho Y^- Y^+] , \tag{11}$$

where ρ is system's density operator. It can be written as

$$\langle Y^- Y^+\rangle = \frac{W_2(\omega_d)}{\Gamma_{0,2}} (|Y_{0,2}|^2 + |Y_{1,2}|^2) , \tag{12}$$

where $Y_{j,k} = \langle E_j|Y^+|E_k\rangle$. In comparision with $Y_{0,2}$, the matrix element $Y_{1,2}$ is almost negligible.

Density matrix approach

Here we derive the two-photon emission rate of the three-component system described in the previous section, by using a perturbative master equation approach. We start from the master equation for the coupled system in the basis of its eigenstates (dressed-states approach), including the external drive (that is the perturbation):

$$\dot{\rho} = i[\rho, H_0 + H_1(t)] + \mathcal{L}[\rho] , \tag{13}$$

where $H_0 = \sum_n \omega_n |E_n\rangle\langle E_n|$ is the diagonalized Hamiltonian of Supplementary Eq. (2). The external driving field is described by the time-dependent interaction

$$H_1(t) = \Omega \cos(\omega_d t)(Y^- + Y^+) , \tag{14}$$

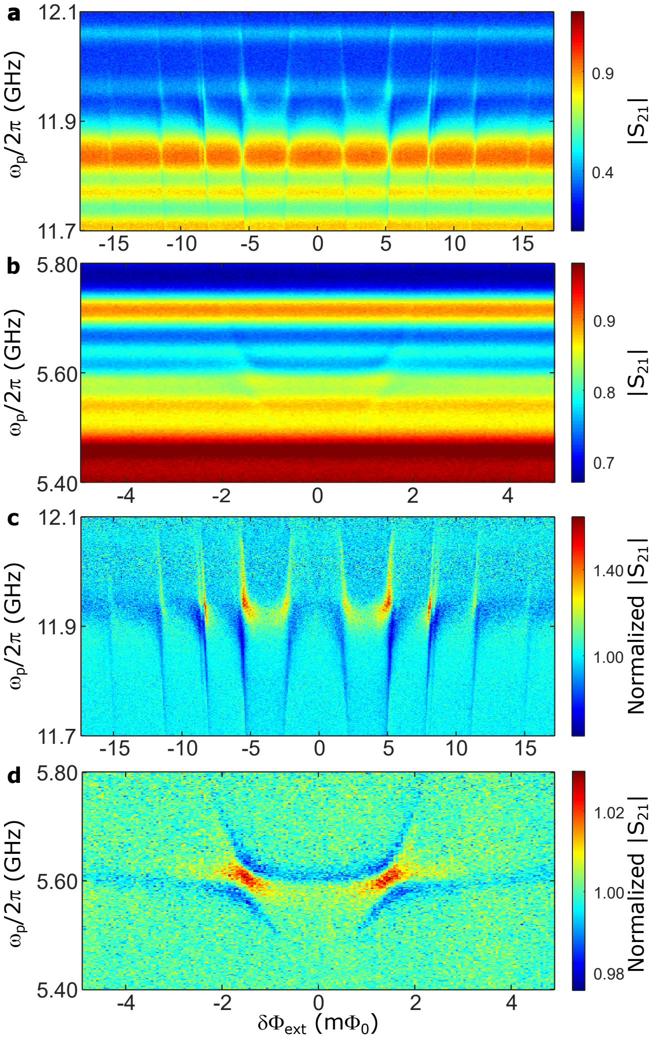

Supplementary Fig. 2. **a**(**c**) and **b**(**d**) are bare (normalized) reflection spectra without the fitting curves of Figs. 2a and 2b in the main text. Source data are provided as a Source Data file.

and $\mathcal{L}[\rho]$ is the dissipator expressed in the eigenstates of the DSC system (see, e.g., Ref. 2). Applying the rotating-wave approximation, we can write

$$H_1(t) = (\Omega/2)(Y^- e^{-i\omega_d t} + Y^+ e^{i\omega_d t})\,. \tag{15}$$

We will also consider an expansion of the density matrix in terms of powers of the drive amplitude, so that we can separate each contribution, i.e.

$$\rho = \rho^{(0)} + \rho^{(1)} + \rho^{(2)} + \ldots = \sum_{n=0}^{\infty} \rho^{(n)}\,. \tag{16}$$

Moreover, since the drive can be expanded in Fourier components as

$$H_1(t) = \sum_k H^{(1)}_{\omega_k} \exp(i\omega_k t)\,, \tag{17}$$

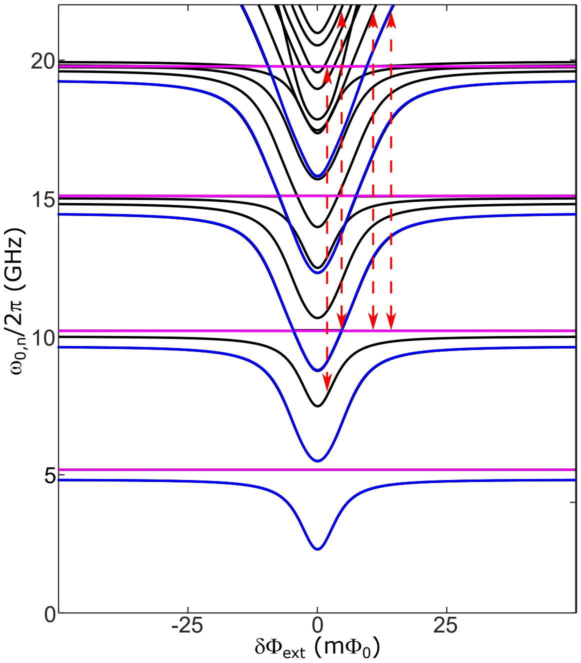

Supplementary Fig. 3. Caculated excitation spectra of the total system ($\omega_{0,n}$). The parameters used in the caculation are the same as in the main text. The dressed excitation energy levels of the deep-strongly coupled qubit–resonator system ω_{0n} (Xmon $\omega_{0n,X}$) are in blue (magenta). The black levels correspond to multiple excitation levels $\omega_{0n} + \omega_{0m,X}$. The red dashed double-arrowed lines indicate the observed sideband transitions in Fig. 2a of the main text.

(actually, we will use here only a single-tone drive) the same can be done with $\rho^{(n)}$, leading to the expression

$$\rho = \sum_{n=0}^{\infty} \sum_k \rho^{(n)}_{\omega_k} \exp(i\omega_k t)\,, \tag{18}$$

where $\omega_k = \pm k\,\omega_d$, with k being an integer number. Considering the steady state, plugging Supplementary Eq. (18) into the master equation, we obtain a set of closed equations separated in driving orders and Fourier components, that at the lowest order looks like:

$$\dot{\rho}^{(0)} = 0 = i[\rho^{(0)}, H_0] + \mathcal{L}[\rho^{(0)}]\,, \tag{19}$$

which leads to the thermal density operator

$$\rho^{(0)} = \frac{1}{\mathcal{Z}} \sum_j \exp\left(-\frac{\hbar\omega_j}{k_B T}\right) |E_j\rangle\langle E_j|\,, \tag{20}$$

with the partition function $\mathcal{Z} = \sum_j \exp\left(-\frac{\hbar\omega_j}{k_B T}\right)$. The next two sets of equations for the first- and second-order density matrices can be formally derived from the master

equation, and they have the following forms:

$$\begin{aligned}\dot{\rho}^{(1)}(\omega_k) &= i\omega_k \rho^{(1)}_{\omega_k} \\ &= i[\rho^{(0)}, H^{(1)}_{\omega_k}] + i[\rho^{(1)}_{\omega_k}, H_0] \\ &\quad + \mathcal{L}[\rho^{(1)}_{\omega_k}]\,, \\ \dot{\rho}^{(2)}(\omega_j+\omega_k) &= i(\omega_j+\omega_k)\rho^{(2)}_{\omega_j+\omega_k} \\ &= i[\rho^{(1)}_{\omega_j}, H^{(1)}_{\omega_k}] + i[\rho^{(2)}_{\omega_j+\omega_k}, H_0] \\ &\quad + \mathcal{L}[\rho^{(2)}_{\omega_j+\omega_k}]\,. \end{aligned} \tag{21}$$

The solutions of the above expressions can be derived sequentially, by plugging the already found solution for the previous order. In this way we find for the first- and second-order steady-state density matrices:

$$\begin{aligned}(\rho^{(1)}_{\omega_k})_{n,m} &= \frac{(H^{(1)}_{\omega_k})_{n,m}}{\omega_k - \omega_{n,m} + i\Gamma_{n,m}/2}(\rho^{(0)}_{m,m} - \rho^{(0)}_{n,n})\,, \\ (\rho^{(2)}_{\omega_j+\omega_k})_{n,m} &= \frac{([H^{(1)}_{\omega_j}, \rho^{(1)}_{\omega_k}])_{n,m} + ([H^{(1)}_{\omega_k}, \rho^{(1)}_{\omega_j}])_{n,m}}{\omega_j+\omega_k - \omega_{n,m} + i\Gamma_{n,m}/2}\,. \end{aligned} \tag{22}$$

The decay rates $\Gamma_{n,m}$ are the sum of all the lossy channels involved in the $|E_m\rangle \to |E_n\rangle$ transition and its expression is shown below. For a nonzero temperature reservoir, one has to take care of the modified decay rates for the generic transition $|E_j\rangle \to |E_i\rangle$ (with $j > i$). For the sake of simplicity, we assume that both the artificial atoms and the lumped-element resonator are thermalized at the same temperature. Thus, the rates can be calculated via the Fermi's golden-rule. At zero-temperature, we obtain $\Gamma^0_{i,j} = \gamma^{(q)}_{i,j} + \gamma^{(a)}_{i,j} + \gamma^{(b)}_{i,j}$, where $\gamma^{(q)}_{i,j} = \gamma_q|\langle E_i|\sigma_x|E_j\rangle|^2$, $\gamma^{(a)}_{i,j} = \gamma_a|\langle E_i|(a+a^\dagger)|E_j\rangle|^2$, and $\gamma^{(b)}_{i,j} = \gamma_b|\langle E_i|(b+b^\dagger)|E_j\rangle|^2$. At $T > 0$, the loss rates become

$$\Gamma_{i,j} = \sum_{s=\{q,a,b\}} \left(\sum_{k>i} \gamma^{(s)}_{i,k}(1+2\bar{n}_{i,k}) + \sum_{k>j} \gamma^{(s)}_{j,k}(1+2\bar{n}_{j,k}) \right), \tag{23}$$

where $\bar{n}_{i,j}$ is the thermal noise that affects the $|E_j\rangle \to |E_i\rangle$ transition, i.e., $\bar{n}_{i,j} = 1/[\exp(\hbar\omega_{i,j}/k_B T) - 1]$. In the following theoretical analysis, corroborated by the standard working temperature of the DSC system ($T \sim$ 30 mK), we can safely ignore the enhancement induced rates, by choosing the relevant $\bar{n}_{i,j} = 0$.

In our calculations, we stop the perturbative development up to the second order, as the essential physics is fully caught. In particular, we can obtain the single- and two-photon absorption by looking at the polarization of the Xmon as a function of the drive frequency, which can be defined as $\langle P(\omega_d)\rangle = \mathrm{Tr}[-i(b-b^\dagger)\rho(\omega_d)]$. In this formula, we can apply further simplifications by (i) considering the case of a ground state environment ($T = 0$) and (ii) dropping the negligible terms of $\langle P(\omega_d)\rangle$, finding that the leading terms that drive the onset of the sought effect yield

$$|\langle P(\omega_d)\rangle| \simeq |(Y_{2,0}\rho^{(2)}_{0,2} + Y_{0,2}\rho^{(2)}_{2,0})| = 2\Re[Y_{2,0}\rho^{(2)}_{0,2}]\,. \tag{24}$$

Terms like $Y_{2,0}\rho^{(1)}_{0,2} + Y_{0,2}\rho^{(1)}_{2,0}$ represent a constant background as they are far-detuned resonances, and thus they can be ignored. Furthermore, we also find that $|Y_{2,0}| \simeq 1$, as it represents the transition matrix element of the weakly coupled Xmon, from its ground to the first-excited level. Following our perturbative approach, we find that

$$\rho^{(2)}_{0,2} = -\frac{\Omega^2}{2}\frac{Y_{0,1}Y_{1,2}}{(\omega_d - \omega_{0,1} + i\Gamma_{0,1}/2)(2\omega_d - \omega_{0,2} + i\Gamma_{0,2}/2)}\,. \tag{25}$$

Finally, as the detected field has frequency $\omega_d \sim \frac{1}{2}\omega_{2,0}$, the non-resonant part of the denominator can be expanded in series, leading to the final expression:

$$|\langle P(\omega_d)\rangle| = \frac{\Omega^2|Y_{0,1}Y_{1,2}|}{\omega_{1,2} - \omega_{0,1}}\frac{\Gamma_{0,2}|Y_{2,0}|}{(2\omega_d - \omega_{0,2})^2 + \Gamma^2_{0,2}/4}\,. \tag{26}$$

Results

Below we show the numerical results achieved with parameters extrapolated by the experimental data. Namely, we use $\omega_r/2\pi = 4.82\,\mathrm{GHz}$, $\Delta/2\pi = 15\,\mathrm{GHz}$, $g/2\pi = 4.55\,\mathrm{GHz}$, $g'/2\pi \approx g_X/2\pi = 28\,\mathrm{MHz}$, and loss rates for the flux qubit, lumped-element resonator and Xmon are chosen to be $\gamma^{(q)}/2\pi = \gamma^{(a)}/2\pi = \gamma^{(b)}/2\pi = 2\,\mathrm{MHz}$. Also, we consider a thermalized DSC system at a temperature of 30 mK. Our model is able to reproduce the experimental results with a very good agreement. In the simulations, we use a quite strong external field exciting the Xmon, in order to produce a two-photon absorption, and its value in terms of linewidth is $\Omega = 200 \times \gamma^{(b)}$.

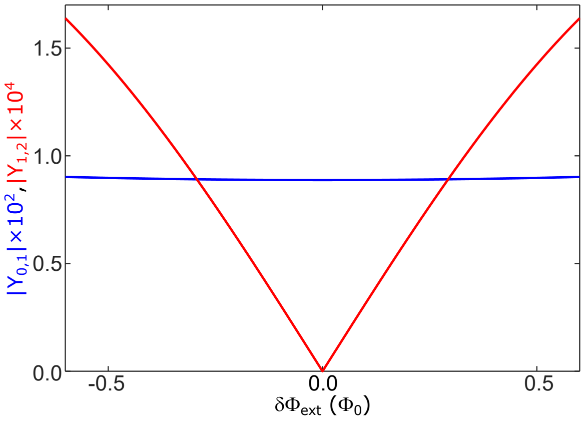

Supplementary Fig. 4. Calculated most relevant terms in the two-photon absorption rate as a function of the external flux bias $\delta\Phi_{\rm ext}$. The detected signal is approximately proportional to the product of these quantities. The calculated $Y_{1,2}$ term goes to zero for $\delta\Phi_{\rm ext} \to 0$, when the parity selection rule for the dressed Xmon is restored. The $Y_{0,2}$ is not shown in the figure as it is almost unity in the whole range investigated here.

Such a value is large enough to ensure the onset of the second-order processes. While for the direct excitation of the single-photon absorption, we reduce Ω by a factor 2×10^6, in doing so we can get the same order of the amplitudes of the single- and two-photon signals, which is consistent with the experimental observation.

Figure 3 in the main text shows the comparison between experimental (top) and theoretical (middle) results calculated for the reported parameters. The left panel shows the weak-excitation, two-tone spectroscopy of the dressed Xmon ($|E_0\rangle \to |E_2\rangle$) transition, while in the right panel, a strong-excitation spectroscopy shows the two-photon resonance at half of the Xmon ($|E_0\rangle \to |E_2\rangle$) transition. Both the experimental and theoretical signals are displayed as a function of the drive frequency ω_d and of the external flux bias, $\delta\Phi_{\rm ext}$. For zero flux bias $\delta\Phi_{\rm ext} = 0$, the two-photon resonance disappears as the parity symmetry is completely restored. The theoretical calculations display the changes in the amplitude of the Xmon polarization $|\langle P(\omega_d)\rangle|$.

Supplementary Fig 4 displays the most relevant term in the two-photon absorption rate as a function of the flux bias $\delta\Phi_{\rm ext}$. It shows the origin of the dependence on the flux bias of the two-photon transition rate for the dressed Xmon. In particular, we observe that the matrix element $Y_{1,2}$ goes to zero for $\delta\Phi_{\rm ext} \to 0$, owing to the restoration of parity symmetry. This disables two-photon transitions from the ground state to the dressed first excited state of the Xmon ($|E_0\rangle \to |E_2\rangle$).

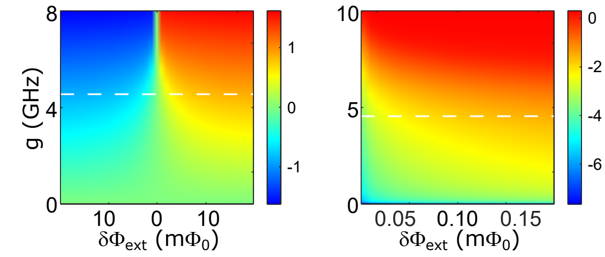

Supplementary Fig. 5. Calculated ground-state coherence $\langle G|a|G\rangle$ of the qubit–resonator system. Left: ground-state coherence $\langle G|a|G\rangle$ versus both the external flux bias $\delta\Phi_{\rm ext}$ and the coupling strength g. The dashed white line represents the measured value of coupling strength in the experiment. Right: as in the left figure, the ground-state coherence $\langle G|a|G\rangle$ is shown only for positive flux bias, and for higher values of the coupling. The scale of the right figure is logarithmic, with a small cutoff to the zero flux bias in order to avoid the log-divergence.

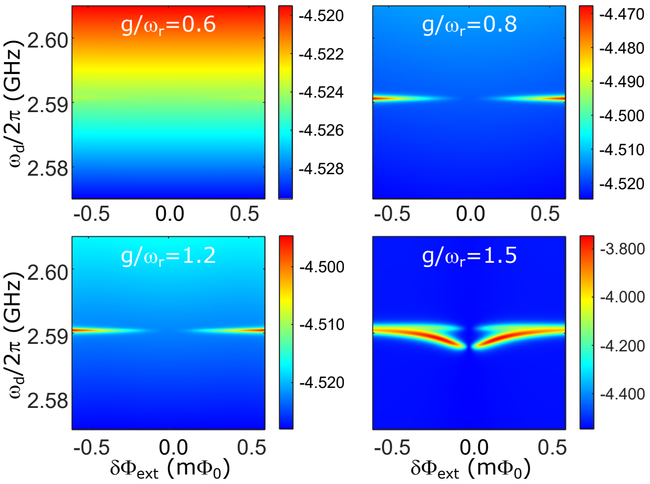

Supplementary Fig. 6. Simulated two-photon spectra of the Xmon versus the effective coupling strength g/ω_r. The negative value of the plot legend is due to the log-scale. The drive intensity applied is the same as in Fig. 3d in the main text.

Further discussions

Supplementary Fig. 5 displays the calculated ground-state coherence $\langle G|a|G\rangle$ of the qubit–resonator system versus both the external flux bias $\delta\Phi_{\rm ext}$ and the coupling strength g. The vacuum expectation value $\langle G|a|G\rangle$ becomes non-negligible when the coupling stength g approaches deep strong and the external flux bias $\delta\Phi_{\rm ext}$ is tuned away from the optimal point.

Supplementary Fig. 6 displays the simulated results of the two-photon spectra of the Xmon versus the effective coupling strength g/ω_r. We can see that when the effective coupling strength g/ω_r is reduced to 0.6, the two-photon signals of the Xmon disappear. For stronger coupling ($g/\omega_r = 1.5$), further features emerge due to

the interaction of the two-photon resonance with other spectral lines.

Symmetry breaking of the quantum vacuum makes the flux-bias-insensitive Xmon experience a *virtual* path to its two-photon resonance. It is not possible if the Xmon is coupled with a normal product-state vacuum (when $g/\omega_r \ll 1$), or when the vacuum symmetry is preserved ($\delta\Phi_{\rm ext} = 0$), in contrast to the cases of flux-tunable qubits, where the two-photon transition becomes allowed naturally when there is a finite external flux bias.

* litf@tsinghua.edu.cn
† salvatore.savasta@unime.it
‡ jqyou@zju.edu.cn

[1] L. Garziano, R. Stassi, A. Ridolfo, O. Di Stefano, and S. Savasta, Vacuum-induced symmetry breaking in a superconducting quantum circuit, Phys. Rev. A **90**, 043817 (2014).

[2] A. Ridolfo, M. Leib, S. Savasta, and M. J. Hartmann, Photon Blockade in the Ultrastrong Coupling Regime, Phys. Rev. Lett. **109**, 193602 (2012).
